# Trends in mental health before and after the onset of the COVID-19 pandemic: a longitudinal survey of a conflict-affected population in Colombia

**DOI:** 10.1186/s13033-024-00621-1

**Published:** 2024-02-05

**Authors:** Rodrigo Moreno-Serra, Sebastian Leon-Giraldo, Nicolas Jater-Maldonado, German Casas, Oscar Bernal

**Affiliations:** 1 Centre for Health Economics, University of York, York, UK; 2 School of Government, Universidad de los Andes, Bogotá, Colombia; 3 Interdisciplinary Centre of Development Studies, Universidad de los Andes, Bogotá, Colombia; 4 Faculty of Medicine, Universidad de los Andes, Bogotá, Colombia; 5 Fundación Santa Fe de Bogotá, Bogotá, Colombia

**Keywords:** Conflict, Health, COVID-19, Mental health, Colombia, SRQ

## Abstract

**Background:**

Focusing on the Meta region in Colombia, we investigated the relationship between mental health, the COVID-19 pandemic, and social determinants of health influenced by over five decades of civil conflict. We studied the post-2016 peace agreement trends in mental health for the population of Meta, before and after the local onset of the pandemic.

**Method:**

We conducted three rounds of a longitudinal health survey in years 2018 with N = 1309 (Women = 709; Men = 600); 2019 with N = 1106 (Women = 597; Men = 509); and 2020 with N = 905 (Women = 499; Men = 406). We measured mental health through the Self-Report Questionnaire (SRQ-20), investigating population trends in the average SRQ score and SRQ-positive frequency (SRQ + , indicating positive tendency towards experiencing mental health disorders).

**Results:**

Between 2018 and 2020, there were reductions in the mean SRQ-20 score by 1.74 points (95% CI -2.30 to -1.18) and in SRQ + frequency by 15 percentage points (95% CI -21.0 to -9.0) for the Meta population. Yet specific subgroups have become more vulnerable to mental illness during the pandemic, for example older age groups (e.g., increase in mean SRQ score among over 60 s by 2.49 points, 95% CI 0.51 to 4.46) and people living with children younger than five years-old (e.g., increase in mean SRQ score by 0.64 points, 95% CI 0.07 to 1.20). Increased mental health vulnerability among specific subgroups may be related to differences in the likelihood of knowing people who tested positive for COVID-19 or died from itf having been in quarantine.

**Conclusion:**

Our findings support the importance of public policies in Colombia (and other low- and middle-income countries) that address the social determinants of mental illness whose influence was likely exacerbated by the pandemic, including persistent job insecurity leading to work and financial pressures, and inadequate support networks for isolated individuals and vulnerable caregivers.

**Supplementary Information:**

The online version contains supplementary material available at 10.1186/s13033-024-00621-1.

## Background

The Director-General of the World Health Organization (WHO) declared the COVID outbreak a public health emergency of international concern (PHEIC) on January 30th, 2020 [1]. As a result, the Colombian government implemented a national lockdown on March 22, 2020, aimed at mitigating the spread of COVID-19 and its impact on the health of the population. Primarily due to its negative economic impact, the national lockdown was lifted on April 25, 2020 [2]. The potential effects of the COVID-19 pandemic and related policies, such as social isolation measures, go beyond harm to physical health and economic activity; they also pose a risk to mental health and well-being, both for people with and without pre-existing mental disorders [3, 4]. Previous analyses suggest that increased social isolation caused by the pandemic has led to deterioration in mental health [5, 6]. In low- and middle-income countries (LMICs), the risks to mental health in the context of the pandemic are increased by social determinants, particularly poverty and pre-existing inequalities in mental health, which may make the impact of the COVID-19 pandemic on mental health even more detrimental than in high-income countries [7].

Our study focuses on Colombia, a middle-income country that provides a highly relevant case study for the (understudied) relationship between the COVID-19 pandemic, social determinants of health, and mental health. Between 1958 and 2020, a civil conflict in Colombia impacted nearly every region of the country, resulting in more than 267,000 deaths, over 4,200 massacres, 8,600 forced disappearances, 15,700 victims of sexual violence, and 7.3 million internally displaced people [8]. Meta, where we conducted our study, was the third Colombian province most affected by the conflict in terms of officially registered victims. In 2016, a peace agreement was signed between the government and the largest rebel armed group, the FARC, which led to significant de-escalation of violence especially in regions where the FARC had a strong prestorically, as in the case of Meta [9, 10].

Nevertheless, after more than five decades of armed conflict, the repercussions of prolonged chronic violence on the mental health of Colombians is significant. More than 40% of adults report exposure to a traumatic event, such as physical mistreatment, sexual abuse, armed conflict, common criminality, having witnessed serious injuries, or the unexpected death of parents or caregivers or suffering from serious illnesses [11]. Individuals directly affected by conflict-related violence, such as internally displaced persons (IDPs), tend to have severe mental health disorders long after the event, particularly high levels of depression and suicide risk [12]. Whilst 10% of the Colombian adult population has a mental health disorder, this prevalence rate is much higher among the large populations directly affected by conflict violence, such as IDPs (56% prevalence) and people living in provinces with high conflict intensity (e.g. 28% prevalence in Meta) [11, 13]. The conflict has also contributed to the deterioration of health infrastructure and health inequities due to its heavier burden on poor and rural citizens, with the populations most affected by violence being particularly vulnerable in both the mental health and healthcare access [10–14].

The COVID-19 pandemic and subsequent social isolation measures in Colombia may have interacted with the aforementioned social determinants of health, to further affect the mental health of conflict-affected populations. However, there is very little research on mental health in the midst of the COVID-19 pandemic that focuses on these vulnerable groups, either in Colombia or in other settings of protracted conflict violence. For Colombia, there are no studies that investigate the issue using individual longitudinal data that would allow comparisons of trends before and after the onset of the pandemic. A cross-sectional study based on an online survey identified higher level of distress among Colombian respondents in March 2020, than among respondents from Brazil, Germany, Israel, Norway and the United States [15]. A cohort study conducted in Tumaco, Colombia found a large increase in the likelihood of anxiety, depression, and stress among caregivers of young children who were displaced during the pandemic or who had pre-existing mental health conditions [16]. In other settings, however, a longitudinal study of 410 Syrian refugees living in the Azraq camp in Jordan found that refugee assessed during the pandemic had less severe PTSD symptoms than those assessed prior to the pandemic [17]. The latter study also found that pre-existing mental health problems were not important predictors of major mental health concerns during the pandemic for this group of refugees.

In summary, while there is evidence highlighting the detrimental mental health consequences of armed conflict for affected communities, including in the long term, there is a lack of evidence about how the mental health of these populations may have been affected by the pandemic context. Our study contributes to the evidence base on this topic by examining post-peace agreement trends in mental health for a representative sample of the conflict-affected population of Meta, Colombia, by comparing periods before and after the local outbreak of the COVID-19 pandemic. We examine the role of several potential predictors of changes in mental health trends during the study period, including the spread of COVID-19 across communities as well as behavioral and economic changes associated to the pandemic. Based on our literature review, our initial hypothesis is that mental health deteriorated in the Meta region after the onset of the COVID-19 pandemic.

## Methods

### Data source

We initially conducted the Conflict, Peace and Health (*Conflicto, Paz y Salud*, CONPAS) survey in 1309 households in the Meta region. The main objective of the survey was to collect information on socioeconomic conditions and health indicators in one of the regions most affected by the armed conflict, following the peace agreement signed in 2016. This effort aimed to facilitate public health analyses in a post-conflict context. The first survey was conducted in 2018, and subsequent rounds were conducted in 2019 and 2020, with the same adult respondents. The 2018 and 2019 interviews were conducted in person between November and December, while the 2020 interviews were conducted by telephone between November 2020 and January 2021. Unfortunately, due to the isolation and quarantine measures being implemented in Colombia at the time, we were unable to interview any individuals in person in 2020. Access to a phone was not a key factor dictating individual non-response to our 2020 survey, however. The 2019 round of the survey included a question about the presence of a cell phone in the interviewee’s home. A total of 1095 of the 1106 individuals surveyed (representing 99.01% of the sample) confirmed having a cell phone in their homes.

**Fig. 1:**
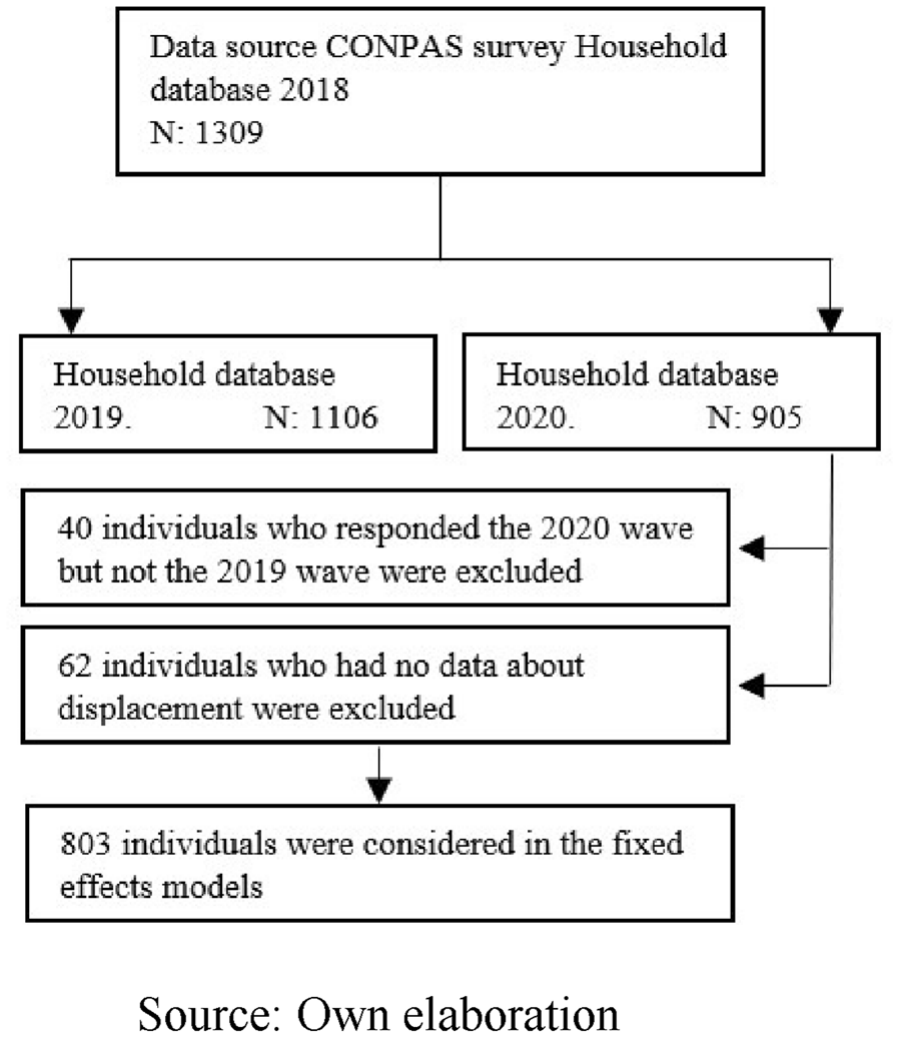
Flowchart showing selection of survey respondents for the statistical analysis

The CONPAS sample selection followed a probabilistic design with stratification to ensure sample representativeness at the level of the total, urban, and rural population of Meta, as well as of the population living in municipalities “heavily”, “lightly” or “not affected” by conflict violence (as per CERAC’s classification of historical persistence and intensity of violence in the municipalities) [18], and in the capital, Villavicencio. Households were selected from blocks identified through multistage sampling. Sampling units were selected using simple random sampling without replacement. Figure 1 shows the data selection process for our analysis. A total of 865 individuals were interviewed in all 3 waves; however, after excluding respondents with missing data, 803 individuals were included in our fixed effects regression analysis.

### Outcomes and independent variables

The WHO developed the Self-Report Questionnaire (SRQ-20), which consists of 20 questions about general mental health and well-being [19]. It is a globally accepted, practical, and well-validated instrument for measuring individual tendencies towards mental health disorders, specifically Common Mental Disorders (CMD) such as depression and anxiety [20], which was included in CONPAS. A person is considered to have a positive tendency towards experiencing mental health disorders if he/she answers "yes" to 8 or more of the 20 questions in the questionnaire. The latter case is referred to in our analysis as SRQ + (a SRQ positive case).

As for the covariates used in our statistical analysis, we extracted information from the 2018 CONPAS wave on gender, ethnicity (defining the majority category as “white” or “mixed”, whereas the minority category includes “black”, “indigenous” and other minority groups), education (highest level of formal education attained), and forced displacement (an indicator variable for displaced status, constructed from a 2018 question about ever having been displaced due to the armed conflict), as a measure of direct exposure to conflict violence. We complement this with an indicator for the persistence/intensity of the armed conflict in the respondent’s municipality of residence [18], as a measure of indirect exposure to conflict.

From all CONPAS waves, we obtained data on the respondent’s place of residence (urban or rural), age group (18–44; 45–60; > 60), marital status (married or in a stable partnership, separated or divorced, widowed and single), and socioeconomic variables. The latter include employment status (formal employee or employer, informal employee or self-employed, economically inactive such as student, unemployed and retired) and total household spending (grouped into quintiles). We also collected information on hospitalization events (indicator variable for whether the respondent had been hospitalized for a health problem in the previous 12 months) as a proxy for physical (ill) health. Finally, we use information on the number of people living in the same household (indicator variable for single person or multi-person household) and the number of children aged 5 years or younger in the household (indicator variable for living with at least one child aged 5 or younger or not living with at least one child aged 5 or younger).

### Statistical analysis

Data analysis was conducted in three phases. The first stage consisted of a descriptive statistical analysis of the 2020 CONPAS data, to obtain a detailed snapshot of the mental health status of our sample during the COVID-19 pandemic. Additionally, we pooled the 2018, 2019 and 2020 CONPAS data to perform a cross-sectional analysis examining the trend in the mean SRQ-20 score over the three years, as well as the proportion of participants with SRQ + in each year. These descriptive analyses included all the respondents in each wave.

In the second phase, we examined the changes in mental health from 2018 to 2020 by estimating fixed-effects regression models (Additional file 1: Appendix S1). The main goals were to measure the changes in mental health trends, between before and after the onset of the pandemic, and how these changes are associated with individual- and household-level covariates. This investigation follows evidence from other countries that some groups, such as women, people living with preschool-age children, lower income groups, the youngest and oldest cohorts, and people with poor physical health, experienced particularly severe deterioration in mental health during the pandemic [5, 21]. All covariates had less than 1% missing data, except for displaced status which had 7.2% missing data. These models included only the balanced longitudinal sample of the 803 respondents who participated in all CONPAS waves and had no missing covariates (Fig. 1).

We estimated two fixed-effects models. In Model 1, we used the individual SRQ-20 score as the dependent variable, i.e. examining increases or decreases in the total score, which ranges from 1 to 20 (where 20 denotes individuals with the worst mental health status as judged by the highest tendency to present a mental health disorder). A positive and statistically significant estimated coefficient for a given covariate from the fixed-effects model indicates that the mean SRQ-20 increased in 2020, compared to 2018, for the particular population subgroup represented by the covariate. This can be interpreted as individuals in that subgroup being, on average, closer to the threshold of exhibiting an SRQ + , although this is not sufficient to infer a worsening of mental health for that subgroup in the period. For the latter, instead, we estimate Model 2 where the dependent variable is a binary indicator for SRQ + , taking the value of one if the respondent has SRQ + and thus a tendency to have a mental health disorder, zero otherwise. In Model 2, a positive and statistically significant coefficient for a given covariate indicates an increase in the probability of SRQ + , i.e. that mental health deteriorated in that subgroup, between 2018 and 2020. The fixed-effects analyses permit an examination of how mental health trends after conflict de-escalation in the Meta region evolved within the context of the COVID-19 pandemic.

In the third phase, using only the cross-sectional CONPAS 2020 data, we estimated five separate linear regression models (through ordinary least-squares estimation) to investigate whether particular demographic and socioeconomic subgroups have had different experiences regarding the spread of COVID-19 in the community, and/or different behavioral or financial changes due to the pandemic, that may help explain any differential changes in mental health indicators across these subgroups during 2018–2020. We used the following CONPAS 2020 questions to construct the five dependent variables analyzed separately:

1. In the last month, did you have symptoms related to COVID-19? (Indicator variable: 1 = yes, 0 = no)
2. How many people do you personally know from outside your household who have tested positive for COVID-19? (Indicator variable: 1 = knows someone, 0 = does not know anyone)
3. How many people did you personally know who have died from COVID-19? (Indicator variable: 1 = knew someone, 0 = did not know anyone)
4. Are you currently in quarantine or self-quarantine? (Indicator variable: 1 = yes, 0 = no)
5. How important is the threat imposed by the pandemic-related measures and restrictions to your household’s finances? (Indicator variable: 1 = severe or moderate, 0 = not important or it is not a threat at all)

The covariates used in each of the five models above were the same as those used in the fixed-effects models. All estimations were performed with robust standard errors using Stata version 17.

### Patient and public involvement

Various technical consultations with representatives of civil society groups from Meta and other regions (including patient and conflict victim associations), as well as Meta and national government health authorities, took place between 2017 and 2020. Residents of Meta municipalities were involved in content validity testing and provided feedback on the CONPAS survey instrument.

## Results

In 2020, the mean SRQ-20 score was 4.6 (95% CI 4.2 to 4.9) with 24.6% (21.8 to 27.4) of participants exceeding the threshold score indicative of a tendency to present mental health disorders (Table 1). Women had a higher proportion of SRQ + cases at 26.5% (95% CI 26.4 to 26.5) compared to men at 22.4% (22.3 to 22.5). People over 60 years of age had a higher proportion of SRQ + cases (29.3%; 95% CI 26.4 to 32.3) than younger people. Ethnic minorities had a higher proportion of SRQ + cases (31.4%; 95% CI 31.3 to 31.5). Quintiles 1 and 5 of household expenditures concentrated the greatest proportions of SRQ + cases. The proportion of SRQ + cases tended to be higher also among people who had been admitted to hospital in the previous 12 months, those living in municipalities that were heavily affected by the armed conflict, and those displaced by the conflict. Regarding the variables related to COVID, we found that, among the 905 total respondents, 69 (7.6%) individuals reported experiencing symptoms within the last month. Among these symptomatic individuals, only 26 (37.68%) had a COVID-19 test.

**Table 1:** Descriptive statistics—2020 CONPAS survey wave

	Sample	Mean SRQ score (95% CI)	SRQ +	SRQ + frequency (95% CI)
Overall	905 (100%)	4.6 (4.2–4.9)	223	24.6% (21.8–27.4)
Gender				
Men	406 (44.9%)	4.1 (3.6–4.5)	91	22.4% (19.7–25.1)
Women	499 (55.1%)	5.0 (4.5–5.4)	132	26.5% (23.6–29.3)
Age group				
18–44	363 (40.1%)	3.8 (3.3–4.2)	70	19.3% (16.7–21.9)
45–60	317 (35.0%)	4.9 (4.3–5.4)	87	27.4% (24.5–30.4)
> 60	225 (24.9%)	5.5 (4.8–6.1)	66	29.3% (26.4–32.3)
Household expenditure quintile				
1 (Lowest)	181 (20.0%)	5.3 (4.5–6.0)	53	29.3% (26.3–32.2)
2	207 (22.9%)	4.5 (3.9–5.2)	52	25.1% (22.3–27.9)
3	195 (21.5%)	3.6 (3.0–4.2)	34	17.4% (15.0–19.9)
4	143 (15.8%)	4.3 (3.6–5.1)	33	23.1% (20.3–25.8)
5 (Highest)	179 (19.8%)	5.1 (4.3–5.9)	51	28.5% (25.6–31.4)
Employment status				
Formal employee or employer	114 (12.6%)	3.8 (3.0–4.6)	25	21.9% (19.2–24.6)
Informal employee or self-employed	384 (42.4%)	4.2 (3.7–4.6)	83	21.6% (18.9–24.3)
Inactive	364 (40.2%)	5.2 (4.6–5.7)	103	28.3% (25.4–31.2)
Unemployed	29 (3.2%)	6.5 (4.5–8.6)	11	37.9% (34.8–41.1)
Retired	14 (1.5%)	2.6 (1.4–3.9)	1	7.1% (5.5–8.8)
Ethnicity^⁋^				
Majority	714 (78.9%)	4.4 (4.1–4.8)	163	22.8% (20,1–25,6)
Minority	191 (21.1%)	5.1 (4.4–5.9)	60	31.4% (28,4–34,4)
Marital status^⁋^				
Married	200 (22.1%)	4.4 (3.7–5.1)	42	21.0% (18.3–23.7)
Stable partnership	384 (42.4%)	4.1 (3.6–4.6)	88	22.9% (20.2–25.7)
Separated or divorced	201 (22.2%)	5.1 (4.4–5.8)	58	28.9% (25.9–31.8)
Widower	64 (7.1%)	6.4 (5.1–7.8)	21	32.8% (29.8–35.9)
Single	56 (6.2%)	4.4 (3.1–5.7)	14	25.0% (22.2–27.8)
Education level^⁋^				
No formal education	61 (6.7%)	6.7 (5.4–8.1)	26	42.6% (39.4–45.9)
Preschool or primary	383 (42.3%)	5.5 (5.0–6.0)	117	30.5% (27.5–33.6)
High school	292 (32.3%)	3.4 (2.9–3.9)	47	16.1% (13.7–18.5)
Higher education	169 (18.7%)	3.7 (3.0–4.4)	33	19.5% (16.9–22.1)
Area of residence				
Rural	392 (43.3%)	4.4 (3.9–4.9)	93	23.7% (21.0–26.5)
Urban	513 (56.7%)	4.7 (4.3–5.1)	130	25.3% (22.5–28.2)
Hospitalization in the previous 12 months				
No	822 (90.8%)	4.4 (4.0–4.7)	191	23.2% (23.1–23.3)
Yes	83 (9.2%)	6.7 (5.3–8.0)	32	38.6% (38.4–38.7)
Child aged 5 or younger in the household				
No	667 (73.7%)	4.8 (4.4–5.2)	169	25.3% (22.5–28.2)
Yes	238 (26.3%)	3.9 (3.3–4.5)	54	22.7% (20.0–25.4)
Number of people in the household				
One person	71 (7.8%)	5.0 (3.9–6.1)	20	28.2% (25.2–31.1)
More than one person	834 (92.2%)	4.5 (4.2–4.9)	203	24.3% (21.5–27.1)
Conflict intensity in the municipality of residence^⁋^				
	(n = 889)		(n = 219)	
Not affected	214 (24.1%)	4.0 (3.3–4.6)	46	21.5% (18.8–24.2)
Villavicencio	172 (19.3%)	4.3 (3.6–5.0)	39	22.7% (19.9–25.4)
Lightly affected	279 (31.4%)	4.8 (4.2–5.4)	71	25.4% (22.6–28.3)
Heavily affected	224 (25.2%)	5.1 (4.4–5.8)	63	28.1% (25.2–31.1)
Displaced^⁋^				
	(n = 842)		(n = 213)	
No	441 (48.7%)	3.5 (3.2–3.9)	72	16.3% (13.9–18.7)
Yes	401 (44.3%)	5.9 (5.4–6.4)	141	35.2% (32.1–38.3)
COVID-19 VARIABLES				
In the last month, did you have symptoms related to COVID-19?				
Yes	69 (7.6%)	7.5 (6.1–9)	32	46.4% (43.1–49.6)
No	836 (92.4%)	4.3 (4–4.6)	191	22.8% (20.1–25.6)
If you had symptoms in the last month, did you take a COVID-19 test?				
Yes	26 (37.7%)	6.4 (4.3–8.4)	9	34.6% (23.4–45.8)
No	43 (62.3%)	8.2 (6.2–10.2)	23	53.5% (41.7–65.3)

^⁋^Measured during the 2018 CONPAS wave

Table 2 shows that the proportion of participants with a significant tendency to have a mental disorder decreased between 2018 (32.4%; 95% CI 29.9 to 34.9) and 2020 (24.6%; 95% CI 21.8 to 27.4). In 2018, women had about twice as high SRQ + case frequency (41.9%; 95% CI 39.2 to 44.6) than men (21.2%; 95% CI 19.0 to 23.4). By 2020, the SRQ + case frequency decreased significantly for women (26.5%; 95% CI 23.6 to 29.3), but not for men. Other noteworthy changes during the 2018–2020 period were reductions in the frequency of SRQ + cases for individuals living in all areas defined according to conflict intensity, and for both displaced and non-displaced individuals.

**Table 2:** Mean SRQ-20 scores and frequency of participants with SRQ + in the CONPAS survey, by year (2018–2020)

	Mean SRQ score (95% CI)			SRQ + frequency (95% CI)		
	2018 (N = 1309)	2019 (N = 1106)	2020 (N = 905)	2018 (N = 1309)	2019 (N = 1106)	2020 (N = 905)
Overall	5.7(5.5–6.0)	5.1 (4.8–5.4)	4.6 (4.2–4.9)	32.4% (29.9–34.9)	28.5% (25.8–31.1)	24.6% (21.8–27.4)
Gender						
Men	4.5 (4.2–4.9)	4.2 (3.8–4.6)	4.1 (3.6–4.5)	21.2% (19.0–23.4)	21.8% (19.4–24.2)	22.4% (19.7–25.1)
Women	6.7 (6.3–7.1)	5.9 (5.5–6.3)	5.0 (4.5–5.4)	41.9% (39.2–44.6)	34.2% (31.4–37.0)	26.5% (23.6–29.3)
Age group						
18–44	4.9 (4.5–5.3)	4.6 (4.1–5.0)	3.8 (3.3–4.2)	26.9% (24.5–29.3)	24.8% (22.2–27.3)	19.3% (16.7–21.9)
45–60	6.0 (5.6–6.5)	5.3 (4.8–5.9)	4.9 (4.3–5.4)	35.1% (32.5–37.7)	30.0% (27.3–32.7)	27.4% (24.5–30.4)
> 60	7.0 (6.4–7.5)	5.7 (5.1–6.3)	5.5 (4.8–6.1)	39.9% (37.2–42.5)	32.6% (29.8–35.4)	29.3% (26.4–32.3)
Household expenditure quintile						
1 (Lowest)	6.3 (5.7–6.8)	5.0 (4.4–5.5)	5.3 (4.5–6.0)	36.6% (34.0–39.2)	25.6% (23.0–28.1)	29.3% (26.3–32.2)
2	5.3 (4.8–5.9)	4.6 (3.8–5.5)	4.5 (3.9–5.2)	29.1% (26.6–31.5)	23.9% (21.4–26.4)	25.1% (22.3–27.9)
3	5.3 (4.5–6.0)	5.2 (4.6–5.9)	3.6 (3.0–4.2)	29.8% (27.3–32.3)	29.3% (26.6–32.0)	17.4% (15.0–19.9)
4	5.5 (5.0–6.1)	5.1 (4.4–5.7)	4.3 (3.6–5.1)	29.7% (27.2–32.2)	30.4% (27.7–33.1)	23.1% (20.3–25.8)
5 (Highest)	6.0 (5.4–6.7)	5.6 (4.9–6.3)	5.1 (4.3–5.9)	36.4% (33.8–39.1)	33.3% (30.6–36.1)	28.5% (25.6–31.4)
Employment status						
Formal employee or employer	4.9 (4.3–5.5)	4.4 (3.6–5.1)	3.8 (3.0–4.6)	25.8% (23.4–28.2)	23.6% (21.1–26.1)	21.9% (19.2–24.6)
Informal employee or self-employed	5.3 (4.9–5.7)	4.9 (4.5–5.3)	4.2 (3.7–4.6)	29.0% (26.6–31.5)	26.8% (24.2–29.4)	21.6% (18.9–24.3)
Inactive	6.8 (6.3–7.3)	5.7 (5.2–6.2)	5.2 (4.6–5.7)	40.9% (38.2–43.5)	33.2% (30.5–36.0)	28.3% (25.4–31.2)
Unemployed	4.8 (3.5–6.0)	6.0 (4.2–7.8)	6.5 (4.5–8.6)	27.3% (24.9–29.7)	34.4% (31.6–37.2)	37.9% (34.8–41.1)
Retired	5.3 (3.6–6.9)	4.6 (2.8–6.4)	2.6 (1.4–3.9)	29.4% (26.9–31.9)	21.4% (19.0–23.8)	7.1% (5.5–8.8)
Ethnicity						
Majority	5.6 (5.3–5.9)	4.9 (4.6–5.2)	4.4 (4.1–4.8)	31.4% (28.8–33.9)	26.6% (24.0–29.2)	22.8% (20.1–25.6)
Minority	6.2 (5.7–6.8)	5.8 (5.2–6.5)	5.1 (4.4–5.9)	36.2% (33.6–38.8)	35.2% (32.4–38.1)	31.4% (28.4–34.4)
Marital status						
Married	5.5 (5.0–6.1)	4.7 (4.1–5.3)	4.4 (3.7–5.1)	29.5% (27.1–32.0)	26.8% (24.2–29.4)	21.0% (18.3–23.7)
Stable partnership	5.2 (4.8–5.6)	4.9 (4.5–5.3)	4.1 (3.6–4.6)	28.5% (26.1–31.0)	27.0% (24.4–29.6)	22.9% (20.2–25.7)
Separated or divorced	6.6 (6.1–7.2)	5.5 (4.8–6.3)	5.1 (4.4–5.8)	39.1% (36.5–41.8)	32.8% (30.1–35.6)	28.9% (25.9–31.8)
Widower	7.7 (6.6–8.7)	6.7 (5.6–7.8)	6.4 (5.1–7.8)	52.1% (49.4–54.8)	34.5% (31.7–37.3)	32.8% (29.8–35.9)
Single	4.2 (3.4–5.0)	4.9 (3.9–6.0)	4.4 (3.1–5.7)	22.2% (20.0–24.5)	25.3% (22.7–27.9)	25.0% (22.2–27.8)
Education level						
No formal education	7.8 (6.7–8.9)	6.0 (5.4–6.6)	6.7 (5.4–8.1)	44.3% (41.6–47.0)	33.2% (30.4–36.0)	42.6% (39.4–45.8)
Preschool or primary	6.6 (6.2–7.0)	5.3 (4.8–5.8)	5.5 (5.0–6.0)	39.3% (36.6–41.9)	29.8% (27.1–32.5)	30.5% (27.5–33.5)
High school	4.9 (4.5–5.4)	4.6 (4.0–5.1)	3.4 (2.9–3.9)	26.9% (24.5–29.3)	26.6% (24.0–29.2)	16.1% (13.7–18.5)
Higher education	4.6 (4.1–5.1)	4.0 (3.3–4.7)	3.7 (3.0–4.4)	23.8% (21.5–26.1)	20.9% (18.5–23.3)	19.5% (16.9–22.1)
Area of residence						
Rural	5.7 (5.3–6.1)	5.1 (4.7–5.6)	4.4 (3.9–4.9)	29.8% (27.3–32.3)	28.1% (25.5–30.8)	23.7% (21.0–26.5)
Urban	5.7 (5.4–6.1)	5.1 (4.7–5.5)	4.7 (4.3–5.1)	34.1% (31.6–36.7)	28.8% (26.1–31.4)	25.3% (22.5–28.2)
Hospitalization in the previous 12 months						
No	5.4 (5.1–5.6)	4.8 (4.5–5.2)	4.4 (4.0–4.7)	29.7% (27.3–32.2)	26.4% (23.8–29.0)	23.2% (20.5–26.0)
Yes	8.1 (7.3–8.8)	7.0 (6.2–7.9)	6.7 (5.3–8.0)	51.2% (48.5–53.9)	43.7% (40.8–46.6)	38.6% (35.4–41.7)
Child aged 5 or younger in the household						
No	5.6 (5.1–6.2)	5.2 (4.8–5.5)	4.8 (4.4–5.2)	31.2% (28.7–33.7)	29.2% (26.5–31.9)	25.3% (22.5–28.2)
Yes	5.7 (5.4–6.0)	5.0 (4.4–5.6)	3.9 (3.3–4.5)	32.7% (30.2–35.3)	26.4% (23.8–29.0)	22.7% (20.0–25.4)
Number of people in the household						
One person	5.1 (4.4–5.7)	4.9 (4.2–5.7)	5.0 (3.9–6.1)	25.7% (23.4–28.1)	24.8% (22.3–27.4)	28.2% (25.2–31.1)
More than one person	5.8 (5.5–6.1)	5.1 (4.8–5.5)	4.5 (4.2–4.9)	33.4% (30.8–35.9)	29.0% (26.3–31.7)	24.3% (21.5–27.1)
Conflict intensity in the municipality of residence						
		(N = 1100)	(N = 889)		(N = 1100)	(N = 889)
Not affected	5.3 (4.8–5.8)	4.8 (4.2–5.4)	4.0 (3.3–4.6)	29.6% (27.1–32.1)	25.7% (23.1–28.3)	21.5% (18.8–24.2)
Villavicencio	5.3 (4.8–5.9)	5.1 (4.5–5.8)	4.3 (3.6–5.0)	31.3% (28.8–33.8)	29.5% (26.8–32.2)	22.7% (19.9–25.4)
Lightly affected	5.9 (5.5–6.4)	5.3 (4.8–5.9)	4.8 (4.2–5.4)	33.7% (31.2–36.3)	29.6% (26.9–32.3)	25.4% (22.6–28.3)
Heavily affected	6.2 (5.6–6.7)	5.0 (4.4–5.6)	5.1 (4.4–5.8)	34.3% (31.7–36.9)	28.2% (25.5–30.9)	28.1% (25.2–31.1)
Displaced						
	(N = 1213)	(N = 1020)	(N = 842)			
No	4.9 (4.5–5.2)	4.4 (4.0–4.8)	3.5 (3.2–3.9)	25.7% (23.2–28.2)	23.7% (21.1–26.3)	16.3% (13.8–18.8)
Yes	6.9 (6.5–7.4)	6.2 (5.7–6.6)	5.9 (5.4–6.4)	42.3% (39.5–45.1)	36.0% (33.1–39.0)	35.2% (31.9–38.4)

Overall, there was a significant reduction in the mean SRQ-20 score for the Meta population from 2018 to 2020, according to our fixed effects estimations (Table 3). The results for Model 1 show that the mean SRQ-20 score in 2019 decreased by -0.82 (95% CI − 1.34 to − 0.31) points compared to 2018, while the mean SRQ-20 score in 2020 was -1.74 (95% CI − 2.30 to − 1.18) points lower than in 2018. In other words, on average, respondents tended to move away from the SRQ-20 threshold that indicates a risk of mental disorder. Model 2, which presents the results with SRQ + as the dependent variable, shows a similar trend. In 2019, the frequency of SRQ + cases decreased by 5 percentage points (95% CI − 11.0 to 1.0) on average compared to 2018, and in 2020 this reduction reached 15 percentage points (95% CI − 21.0 to − 9.0) compared to 2018. Taken together, the evidence suggests that the downward trend in the mean SRQ-20 score and SRQ + cases in the region continued even during the COVID-19 pandemic.

**Table 3:** Fixed-effects regression analysis

	MODEL 1 (N = 803) SRQ score		MODEL 2 (N = 803) SRQ +	
	Coefficient (95% CI)	P > t	Coefficient (95% CI)	P > t
Year				
2018 (Base category)				
2019	− 0.82 (− 1.34; − 0.31)	0.0017	− 0.05 (− 0.11; 0.01)	0.0789
2020	− 1.74 (− 2.30; − 1.18)	0.0000	− 0.15 (− 0.21; − 0.09)	0.0000
Interaction term: Gender*Year				
Women in 2018 (Base category)				
Preexisting gap (Men*2018)	− 2.00 (− 2.76; − 1.24)	0.0000	− 0.19 (− 0.27; − 0.11)	0.0000
Men*2019	0.49 (− 0.12; 1.11)	0.1141	0.08 (0.01; 0.15)	0.0207
Men*2020	0.81 (0.19; 1.44)	0.0112	0.14 (0.07; 0.21)	0.0001
Age group				
18–44 (Base category)				
45–60	1.32 (0.02; 2.62)	0.0461	0.07 (− 0.08; 0.21)	0.3717
> 60	2.49 (0.51; 4.46)	0.0136	0.20 (− 0.04; 0.43)	0.0978
Household expenditure quintile				
Quintile 1 (Lowest, base category)				
2	0.17 (− 0.40; 0.75)	0.5497	0.00 (− 0.05; 0.06)	0.8937
3	0.09 (− 0.43; 0.61)	0.7358	0.01 (− 0.05; 0.07)	0.7086
4	0.53 (− 0.06; 1.13)	0.0761	0.03 (− 0.03; 0.09)	0.3074
5 (Highest)	0.83 (0.13; 1.53)	0.0210	0.06 (− 0.01; 0.14)	0.0875
Employment status				
Formal employee or employer (Base category)				
Informal employee or self-employed	− 0.36 (− 1.12; 0.39)	0.3486	− 0.05 (− 0.13; 0.03)	0.2158
Inactive	0.16 (− 0.67; 0.98)	0.7096	− 0.01 (− 0.10; 0.07)	0.7546
Unemployed	1.13 (− 0.01; 2.26)	0.0510	0.03 (− 0.10; 0.15)	0.6694
Retired	1.92 (− 0.07; 3.90)	0.0581	0.20 (− 0.07; 0.48)	0.1471
Interaction term: Ethnicity*Year				
Majority in 2018 (Base category)				
Preexisting gap (Minority*2018)	0.27 (− 0.48; 1.02)	0.7000	0.00 (− 0.07; 0.09)	0.8313
Minority*2019	0.49 (− 0.35; 1.32)	0.2518	0.06 (− 0.03; 0.15)	0.1664
Minority*2020	0.41 (− 0.36; 1.18)	0.3000	0.09 (0.01; 0.17)	0.0198
Marital status				
Married or stable partnership (Base category)				
Separated or divorced	0.78 (− 0.20; 1.77)	0.1196	0.09 (− 0.01; 0.19)	0.0824
Widower	− 0.28 (− 1.98; 1.42)	0.7463	0.05 (− 0.14; 0.24)	0.6126
Single	0.11 (− 1.25; 1.47)	0.8784	0.00 (− 0.14; 0.14)	0.9584
Education level				
No formal education (Base category)				
Preschool or primary	− 0.19 (− 0.88; 0.50)	0.5869	0.00 (− 0.07; 0.07)	0.9832
High school	− 0.40 (− 1.54; 0.74)	0.4908	− 0.02 (− 0.14; 0.10)	0.7479
Higher education	− 0.30 (− 1.75; 1.15)	0.6836	− 0.06 (− 0.22; 0.10)	0.4538
Area of residence				
Rural (Base category)				
Urban	− 0.43 (− 1.46; 0.59)	0.4040	− 0.08 (− 0.17; 0.02)	0.1335
Hospitalization in the previous 12 months				
No (Base category)				
Yes	0.91 (0.32; 1.51)	0.0026	0.05 (− 0.01; 0.11)	0.0792
Child aged 5 or younger in the household				
No (Base category)				
Yes	0.64 (0.07; 1.20)	0.0269	0.06 (0.00; 0.12)	0.0438
Number of people in the household				
One person (Base category)				
More than one person	0.34 (− 0.64; 1.31)	0.4964	− 0.02 (− 0.11; 0.07)	0.6551
Conflict intensity in the municipality of residence				
Not affected (Base category)				
Villavicencio	1.99 (− 1.50; 5.49)	0.2637	0.02 (− 0.34; 0.39)	0.8947
Lightly affected	− 0.19 (− 3.22; 2.85)	0.9044	− 0.18 (− 0.47; 0.11)	0.2266
Heavily affected	0.05 (− 3.01; 3.12)	0.9728	− 0.22 (− 0.55; 0.11)	0.1915
Interaction term: Displaced*Year				
Not displaced in 2018 (Base category)				
Preexisting gap (Displaced*2018)	1.96 (1.31; 2.60)	0.0000	0.18 (0.12; 0.25)	0.0000
Displaced*2019	− 0.30(− 0.95; 0.34)	0.3563	− 0.05 (− 0.12; 0.02)	0.1280
Displaced*2020	0.06 (− 0.60; 0.72)	0.8658	− 0.01 (− 0.08; 0.06)	0.6900
Constant	4.04 (1.21; 6.88)	0.0053	0.406 (0.126; 0.687)	0.0046

The difference between the mean SRQ scores of women and men decreased significantly between 2018 and 2020. In 2020, the mean SRQ score for men increased by an additional 0.81 (95% CI 0.19 to 1.44) points compared to the change observed for women, relative to the baseline. That is, mean SRQ scores increased more for men between 2018 and 2020, resulting in a reduction of the preexisting SRQ gap between men and women (which favored men at the 2018 baseline) of − 2.00 points (95% CI − 2.76 to − 1.24). The descriptive trends (Table 2) suggest that this reduction in the gap between women’s and men’s SRQ scores is likely due mainly to a reduction in women’s mean SRQ score over the 2018–2020 period, including during the pandemic period, although women still tended to present (on average) higher SRQ scores than men in 2020. The same picture is suggested by the results of the SRQ + analysis. The frequency of SRQ + cases among men rose by 14 percentage points (95% CI 7.0 to 21.0) in 2020 compared to women at the baseline, reducing markedly the pre-existing men-women gap in SRQ + cases favoring men (by − 19 percentage points; 95% CI − 27.0 to − 11.0). Again, the trends in Table 2 indicate that the reduction in the men-women gap in SRQ + cases between 2018 and 2020 was due to a downward trend in the frequency of SRQ + cases among women throughout the period.

The results in Table 3 show that, on average, SRQ scores increased more for the two older age groups over the period than for adults aged 18–44. Moreover, people aged 60 and over showed an increase of 20 percentage points (95% CI -4.0 to 43.0) in the frequency of SRQ + cases compared to the change observed for 18–44 year olds between 2018 and 2020, although this association is only statistically significant at the 10% level (p = 0.0978). People who had been hospitalized in the previous 12 months showed a greater average increase over the period in both SRQ scores (by 0.91 points; 95% CI 0.32 to 1.51) and frequency of SRQ + cases (by 5 percentage points; 95% CI -1.0 to 11.0, p = 0.0792), than those who had not been hospitalized. People living with at least one child aged 5 years or younger also experienced greater increases on average in both SRQ scores (by 0.64 points; 95% CI 0.07 to 1.20) and frequency of SRQ + cases (by 6 percentage points; 95% CI 0.0 to 12.0) than those living with no child aged 5 or younger.

Regarding other socioeconomic and demographic groups, Table 3 shows that people in the highest quintile of total household expenditure experienced a greater increase in SRQ scores (by 0.83 points on average; 95% CI 0.13 to 1.53) and SRQ + frequency (by 6 percentage points; 95% CI -1.0 to 14.0, p = 0.0875) between 2018 and 2020 than those in the lowest expenditure quintile. SRQ scores also increased more on average for the unemployed (by 1.13 points; 95% CI -0.01 to 2.26, p = 0.051) and the retired (1.92 points; 95% CI -0.07 to 3.90, p = 0.0581) than for formal sector workers or employers. In terms of SRQ + frequency, the latter increased more on average for ethnic minorities (by 9 percentage points; 95% CI 1.0 to 17.0) between 2018–20 compared to the corresponding change for the majority white or mixed ethnicity, with this increase mostly evident in 2020 (i.e. after the pandemic’s onset). There is some evidence of a larger average increase in SRQ + frequency among those who were separated or divorced individuals (by 9 percentage points; 95% CI -1.0 to 19.0, p = 0.0824), compared to the change experienced by individuals who were married or in a stable partnership. Despite pre-existing gaps in 2018 in both SRQ scores (by an average of 1.96 points; 95% CI 1.31 to 2.60) and SRQ + frequency (by 18 percentage points; 95% CI 12.0 to 25.0) that disadvantaged individuals who were displaced by the conflict at some point in their lives, these gaps do not appear to have changed in 2019 or 2020, thus suggesting that displaced individuals did not experience changes in mental health indicators during the period that were different from the changes observed among the non-displaced. Finally, we found no evidence of any differential changes during the period, in SRQ scores or SRQ + frequency, according to educational attainment, area of residence, number of people in the household, or conflict intensity in the municipality of residence.

Because of the dichotomous nature of the SRQ + variable, logistic regression models may be considered a more natural choice for analyzing changes in SRQ + frequency over time. Therefore, we also estimated Model 2 using a conditional fixed-effects logistic regression approach for longitudinal data, with coefficients presented as odds ratios. This alternative set of results, presented in Additional file 1: Appendix S2, confirms the conclusions from the linear fixed-effects estimation of Model 2 in Table 3. For ease of interpretation and comparability with the SRQ score results, we focus our discussion on the linear fixed effects estimation for the SRQ + variable as well.

We now investigate whether some demographic and socioeconomic subgroups may have had different experiences regarding the spread of COVID-19 in the community, and/or different behavioral or financial consequences of the pandemic, that could help explain the differential changes in mental health indicators from 2018 to 2020. Table 4 presents the results of five linear multivariate regression models estimated separately for the dependent variables in each column. The same sample of individuals is used here as in the fixed effects estimations. However, missing data on the COVID-19 questions resulted in a smaller estimation sample of 758 individuals. We focus on the results that are particularly relevant to the statistically significant associations between socioeconomic/demographic factors and SRQ variables identified from the fixed effects estimations.

**Table 4:** Associations between individual characteristics and COVID-related information, CONPAS 2020 survey wave (n = 905)

	Had symptoms related to COVID-19 in the previous month (N = 758)	Knows someone personally from outside the household who tested positive for COVID-19 (N = 758)	Knew someone personally who died from COVID-19 (N = 758)	Was in quarantine or self-quarantine at the time of the survey (N = 758)	Thinks that pandemic-related restrictions impose a severe or moderate threat to their household’s finances (N = 758)
Gender (Base category: Women)					
Men	− 0.02 (0.4558) [− 0.07; 0.03]	0.08 (0.0745) [− 0.01; 0.17]	0.00 (0.9394) [− 0.08; 0.09]	− 0.06 (0.1359) [− 0.15; 0.02]	− 0.04 (0.3331) [− 0.11; 0.04]
Age group (Base category: 18–44)					
45–60	− 0.03 (0.2320) [− 0.08; 0.02]	0.06 (0.1485) [− 0.02; 0.15]	0.10 (0.0098) [0.02; 0.18]	0.01 (0.7320) [− 0.07; 0.09]	− 0.04 (0.2700) [− 0.11; 0.03]
> 60	− 0.02 (0.4456) [− 0.09; 0.04]	0.00 (0.9894) [− 0.11; 0.11]	0.06 (0.2418) [− 0.04; 0.16]	0.14 (0.0118) [0.03; 0.24]	− 0.03 (0.5405) [− 0.12; 0.06]
Household expenditure quintile (Base category: Quintile 1, lowest)					
2	− 0.04 (0.1082) [− 0.09; 0.01]	− 0.06 (0.3346) [− 0.17; 0.06]	0.00 (0.9208) [− 0.10; 0.09]	− 0.09 (0.0871) [− 0.20; 0.01]	0.10 (0.0355) [0.01; 0.18]
3	− 0.02 (0.4371) [− 0.08; 0.03]	0.03 (0.5239) [− 0.07; 0.13]	0.06 (0.1745) [− 0.03; 0.14]	− 0.03 (0.4943) [− 0.13; 0.06]	− 0.04 (0.4063) [− 0.13; 0.05]
4	− 0.06 (0.0412) [− 0.11; 0.00]	0.05 (0.3488) [− 0.06; 0.16]	0.07 (0.1547) [− 0.03; 0.17]	− 0.01 (0.8934) [− 0.11; 0.10]	0.02 (0.6856) [− 0.07; 0.11]
5 (Highest)	− 0.01 (0.8153) [− 0.07; 0.06]	0.19 (0.0008) [0.08; 0.30]	0.22 (0.0001) [0.11; 0.33]	0.00 (0.9808) [− 0.11; 0.11]	0.03 (0.5287) [− 0.06; 0.12]
Employment status (Base category: Formal employee or employer)					
Informal employee or self− employed	− 0.07 (0.1026) [− 0.15; 0.01]	0.02 (0.7426) [− 0.10; 0.14]	0.04 (0.5021) [− 0.08; 0.15]	0.14 (0.0026) [0.05; 0.23]	0.11 (0.0467) [0.00; 0.22]
Inactive	− 0.10 (0.0219) [− 0.19; − 0.01]	0.00 (0.9934) [− 0.14; 0.14]	0.00 (0.9447) [− 0.13; 0.12]	0.30 (0.0000) [0.18; 0.41]	0.05 (0.4323) [− 0.07; 0.17]
Unemployed	− 0.05 (0.4686) [− 0.18; 0.08]	0.07 (0.5193) [− 0.14; 0.28]	0.06 (0.5470) [− 0.14; 0.27]	0.24 (0.0247) [0.03; 0.45]	0.13 (0.1447) [− 0.05; 0.31]
Retired	− 0.14 (0.0021) [− 0.23; − 0.05]	0.31 (0.0131) [0.07; 0.56]	0.15 (0.3448) [− 0.17; 0.47]	0.18 (0.2618) [− 0.13; 0.50]	0.07 (0.6077) [− 0.20; 0.34]
Ethnicity (Base category: Majority, i.e. “white” or “mixed”)					
Minority	0.00 (0.8569) [− 0.05; 0.04]	− 0.05 (0.2232) [− 0.13; 0.03]	0.01 (0.7761) [− 0.06; 0.08]	0.05 (0.2243) [− 0.03; 0.13]	− 0.02 (0.6228) [− 0.09; 0.06]
Marital status (Base category: Married or stable partnership)					
Separated or divorced	0.01 (0.7093) [− 0.04; 0.07]	0.11 (0.0241) [0.01; 0.21]	0.01 (0.8778) [− 0.08; 0.10]	0.05 (0.3225) [− 0.05; 0.14]	0.00 (0.9570) [− 0.08; 0.08]
Widower	− 0.01 (0.7626) [− 0.08; 0.06]	0.11 (0.1895) [− 0.05; 0.27]	0.08 (0.3140) [− 0.08; 0.24]	− 0.05 (0.5449) [− 0.20; 0.11]	− 0.11 (0.1004) [− 0.25; 0.02]
Single	− 0.03 (0.3847) [− 0.10; 0.04]	0.06 (0.4416) [− 0.09; 0.21]	− 0.05 (0.4488) [− 0.18; 0.08]	0.05 (0.4707) [− 0.09; 0.20]	− 0.07 (0.3325) [− 0.21; 0.07]
Education level (Base category: No formal education)					
Preschool or primary	0.00 (0.9689) [− 0.05; 0.05]	0.03 (0.5402) [− 0.06; 0.12]	0.02 (0.6213) [− 0.06; 0.09]	− 0.11 (0.0125) [− 0.20; − 0.02]	0.06 (0.1070) [− 0.01; 0.14]
High school	− 0.01 (0.8528) [− 0.07; 0.06]	0.12 (0.0467) [0.00; 0.23]	0.11 (0.0384) [0.01; 0.21]	− 0.08 (0.1709) [− 0.18; 0.03]	0.00 (0.9218) [− 0.09; 0.10]
Higher education	− 0.01 (0.7787) [− 0.09; 0.07]	0.25 (0.0005) [0.11; 0.38]	0.13 (0.0435) [0.00; 0.25]	− 0.05 (0.4179) [− 0.18; 0.07]	0.02 (0.7923) [− 0.10; 0.13]
Area of residence (Base category: Rural)					
Urban	− 0.02 (0.2722) [− 0.06; 0.02]	0.05 (0.1889) [− 0.02; 0.13]	0.02 (0.5622) [− 0.05; 0.08]	0.01 (0.8522) [− 0.06; 0.08]	− 0.02 (0.512) [− 0.09; 0.04]
Hospitalization in the previous 12 months (Base category: No)					
Yes	0.04 (0.2399) [− 0.03; 0.12]	0.07 (0.2629) [− 0.05; 0.19]	− 0.01 (0.7785) [− 0.12; 0.09]	0.07 (0.2313) [− 0.04; 0.18]	0.07 (0.1340) [− 0.02; 0.17]
Child aged 5 or younger in the household (Base category: No)					
Yes	− 0.03 (0.2511) [− 0.07; 0.02]	− 0.06 (0.1519) [− 0.14; 0.02]	− 0.04 (0.3008) [− 0.11; 0.03]	0.03 (0.5161) [− 0.05; 0.10]	0.03 (0.3696) [− 0.04; 0.10]
Number of people in the household (Base category: One person)					
More than one person	0.02 (0.6161) [− 0.05; 0.09]	0.13 (0.0587) [0.00; 0.27]	0.01 (0.9268) [− 0.12; 0.13]	− 0.02 (0.8206) [− 0.15; 0.12]	− 0.06 (0.3325) [− 0.18; 0.06]
Conflict intensity in the municipality of residence (Base category: Not affected)					
Villavicencio	0.05 (0.1458) [− 0.02; 0.11]	0.04 (0.5100) [− 0.08; 0.15]	0.04 (0.4273) [− 0.06; 0.14]	0.01 (0.8982) [− 0.10; 0.11]	0.00 (0.9516) [− 0.09; 0.10]
Lightly affected	0.01 (0.7582) [− 0.04; 0.06]	− 0.04 (0.3887) [− 0.14; 0.05]	0.04 (0.3169) [− 0.04; 0.13]	− 0.01 (0.8674) [− 0.10; 0.08]	− 0.05 (0.2135) [− 0.13; 0.03]
Heavily affected	0.00 (0.9913) [− 0.05; 0.05]	− 0.14 (0.0070) [− 0.24; − 0.04]	− 0.03 (0.5542) [− 0.11; 0.06]	− 0.05 (0.3151) [− 0.15; 0.05]	− 0.09 (0.0415) [− 0.17; 0.00]
Displaced (Base category: No)					
Yes	− 0.02 (0.4679) [− 0.06; 0.03]	0.05 (0.1874) [− 0.02; 0.12]	0.04 (0.2336) [− 0.03; 0.10]	0.06 (0.0798) [− 0.01; 0.13]	0.07 (0.0299) [0.01; 0.13]
Constant	0.19 (0.0102) [0.04; 0.33]	0.13 (0.2801) [− 0.11; 0.37]	0.00 (0.9678) [− 0.22; 0.23]	0.17 (0.1212) [− 0.05; 0.39]	0.79 (0.0000) [0.58; 0.99]

P− values in parentheses and 95% confidence intervals in square brackets

We found that being a man (p = 0.0745), retired or separated/divorced is associated with a higher likelihood of knowing someone from outside the household who tested positive for COVID-19. People in the 45–60 age group are more likely to have personally known someone who died from COVID-19. Being in the highest quintile of household expenditure is associated with higher odds of both knowing someone who tested positive for COVID-19 and knowing someone who has died from the disease. People aged over 60 years of age and the unemployed were more likely to be in quarantine or self-quarantine at the time of the survey. On the other hand, we found no evidence that people who had been recently hospitalized, those who lived with at least one child aged 5 years or younger, or those who belonged to ethnic minority groups had different experiences during the pandemic, as measured by our indicator of community COVID-19 spread, behavioral changes, or financial consequences of the pandemic.

## Discussion

Our study investigates changes in mental health trends among the conflict-affected population of the Meta region, Colombia, following the de-escalation of the armed conflict after the 2016 peace agreement and into the first year of the COVID-19 pandemic.

Contrary to our hypothesis and the findings of other studies suggesting an increase in mental health disorders during the pandemic [7, 22], our results indicate that the trend of mental health improvement in Meta, observed after the peace agreement, persisted after the local onset of the COVID-19 pandemic. This may be due to the historically high burden of mental disorders among the Meta population as a result of decades of intense conflict violence in the region (cf. e.g., [10]). Living under the protracted threat of violence may lead to individual adaptation through the establishment of coping mechanisms that can provide some protection for mental health in the context of other crises or stressful situations, such as the COVID pandemic, and help prevent further deterioration of (already vulnerable) mental health conditions in these situations. Although our data do not allow us to test this hypothesis explicitly, evidence from the pandemic in other settings, and from our own analyses, suggests its plausibility. For example, an analysis conducted in the Netherlands found that while the study population generally deteriorated during the pandemic, there was no significant increase in symptoms among people who suffered from depression, anxiety or obsessive compulsive disorder [4]. Similarly, Syrian refugees in Jordan who had high levels of depression and anxiety before the pandemic actually exhibited less severe symptoms after the onset of the pandemic [17]. This is consistent with the results from our fixed-effects regression analyses: we found no differential changes in SRQ indicators during the study period for people living in conflict-affected or displaced communities, after the conflict deescalated in the region and into the pandemic period, controlling for other demographic and socioeconomic factors. However, further research is needed to explore the relevance of this adaptation hypothesis in settings of protracted crises during the pandemic.

The generally improving trend in mental health indicators in Meta between 2018 and 2020 seems to have been led by a reduction in mean SRQ-20 scores and SRQ + frequency among women, resulting in a shrinkage of the pre-existing gender gap that disadvantaged women for these indicators. This finding should not lead to the conclusion that the gender gap in mental health has disappeared in the Meta region, or in Colombia more generally. Our 2020 data indicates that women in Meta still had an average SRQ-20 score still 0.9 points higher than men (Table 2), and thus were on average closer to the SRQ + threshold than men. At the national level, the 2015 National Mental Health Survey, which used the SRQ-20 questionnaire, indicated that the prevalence of mental disorders in Colombia among women aged 18–44 was 10.8% (95% CI 9.7–12.1) but for men in the same age group this was 7.9% (95% CI 6.7–9.2); moreover, the prevalence of SRQ + among women aged over 44 years old was 13% (95% CI 11.8–14.6) compared to 8.3% (95% CI 7.0–9.8) for men in the same age group [11]. Thus, women should continue to be considered a vulnerable group for the targeting of protective mental health policies and interventions.

Despite the improving trend of mental health indicators *on average* in Meta, our results indicate that specific subgroups remaining particularly vulnerable to mental illness during the pandemic and in the longer term, as judged by a deterioration of their SRQ indicators during the 2018–2020 period. Some of these detrimental trends may have been influenced by different experiences related to COVID-19 spread in the community and associated responses among these specific subgroups. Our analyses suggest an increase in pre-pandemic age differences in SRQ-20 indicators that discriminate against older age groups. We found a deterioration in SRQ indicators by the end of 2020 among those over 60 years of age and retired, who were also more likely to have been in quarantine/self-quarantine or to have known someone who tested positive for COVID-19. This finding highlights the potentially negative impact of the pandemic on the mental health of older individuals, which may be associated to the risks that the pandemic poses to their physical health and increased isolation, aggravated by technological gaps [21]. Our findings put into perspective the results of other studies suggesting that the long-term effects of the pandemic on mental health may be greater among younger people [23]. Our results also point to the potential importance of family or community support networks in mitigating the sense of isolation increased by pandemic-related measures, such as quarantines, which may affect mental health. The potentially detrimental influence of isolation on mental health during the pandemic appears to be further supported by our finding that those who were separated or divorced experienced a greater increase in SRQ + frequency by 2020 than those who were married or in a stable partnership, while also being more likely to know someone who tested positive for COVID-19. Our finding that those living with a child aged 5 years or younger experienced a negative trend in both SRQ-20 indicators between 2018 and 2020, compared to those not living with children under five years of age, lends some support to the argument that the pandemic has tended to place renewed stress pressures and mental health burden on caregivers [16]. This finding reinforces the need for public policies that provide support networks for vulnerable caregivers (e.g., single parents), not least among conflict-affected populations.

Our results also suggest that that the spread of COVID-19 across communities and the resulting behavioral changes may have influenced the differential deterioration in mental health indicators observed among some other demographic and socioeconomic groups by the end of 2020. Specifically, SRQ indicators worsened relatively more by 2020 for the unemployed, working-age individuals between 45–60 years old, and those in the highest quintile of household expenditure. On the one hand, people in the latter two groups were more likely to know someone who tested positive for COVID-19 or who died from the disease—which may in part reflect greater access to COVID testing services by the economically better-off households and communities [24]. On the other hand, a higher likelihood of having been in quarantine/self-quarantine may help explain the continuing trend of worsening SRQ scores among the unemployed by the end of 2020. The above findings support the importance of public policies that address the social determinants of mental health, the influence of which was likely exacerbated by the pandemic, including persistent job insecurity and loss, as well as the need to seek employment amid a weak economy, a deficient social protection network, and an increased risk of contracting COVID-19. In such a context, initiatives such as wider provision of online job finding services and financial aid (e.g., targeted emergency cash transfers) may ease work and financial pressures, and thus mitigate mental health vulnerabilities [7, 23].

The COVID-19 pandemic has exacerbated the (pre-existing) structural barriers to healthcare access in LMIC settings [25]. For Meta, we found that, on average, SRQ-20 indicators worsened more for people who had been hospitalized in the previous 12 months than for those who had not. One possible reason for this is that access conditions to the health system for those in need of care, whether for chronic physical or mental health problems, have generally worsened because of the substantial burden that COVID-19 patients imposed on the Colombian health system, especially during the early stages of the pandemic [26, 27]. Access conditions during the pandemic may have deteriorated even further for specific populations that were already disadvantaged in terms of access to the health system pre-pandemic, such as ethnic minorities [28]. Indeed, our finding that SRQ + frequency increased more among ethnic minorities in 2020 (compared to 2018) may be related to a relatively greater deterioration in access to health services for ethnic minorities than for the majority of the population during the pandemic.

### Box 1 Summary of key policy recommendations from this study

- Women should continue to be considered a vulnerable group for the targeting of protective mental health policies in Colombia.
- Other vulnerable groups that should be targeted by improved access to mental health policies are the over-60 s, retired individuals and ethnic minorities.
- The implementation of interventions that address the social determinants of mental ill health, especially through improved social protection networks (e.g., wider provision of job finding services and financial aid for conflict-affected groups and unemployed individuals), should be a priority action for public policy in Colombia.
- Another priority area for policy should be the strengthening of community support networks for vulnerable caregivers (e.g., single parents), not least among populations directly affected by conflict violence.

As with any study, our analyses are subject to some data limitations. First, we focus on the case of the Meta region. While useful for local health policy, further studies are needed to assess the external validity of our conclusions at the national level in Colombia and for other national settings, preferably from a multidisciplinary perspective given the multiple social determinants through which the COVID-19 pandemic may affect mental health in vulnerable groups [29]. Second, the SRQ-20 is a screening tool whose scores correlate strongly with the presence and future diagnosis of mental health disorders, yet it does not constitute a clinical diagnosis tool. Therefore, our conclusions should be interpreted as referring to changes in trends of people *at risk* of developing a mental disorder. Third, we use hospitalization events as the available proxy for physical health in our analyses, which could underestimate the burden of ill health among groups that face relatively worse access to hospital care in Meta, particularly rural populations. Fourth, in our analyses of associations between individual characteristics and COVID-related information, one of the dependent variables in the linear regression models is "Knows someone personally from outside the household who tested positive for COVID-19". Yet only 37% of people who experienced COVID symptoms within the last month actually had a COVID test (Table 1), suggesting that there may have been barriers to access to testing in Meta, resulting in a potential underestimation of the importance of knowing someone who was sick or presenting symptoms. However, these COVID-related variables are still relevant to understanding the impact on mental health according to different experiences that occurred during the pandemic. Finally, there was non-negligible loss-to-follow-up between our survey rounds: while the 2018 baseline data were collected from 1309 respondents, the fixed-effects models considered a final balanced panel of 803 individuals who participated in all CONPAS rounds and had the complete information set for the variables used in our study. However, comparisons of the 2018 SRQ-20 score means and SRQ + case frequencies between individuals who were lost and those who remained in our sample reveal no statistically significant differences, thus offering reassurance that data have not been selectively lost (Additional file 1: Appendix S3).

Despite data limitations, our study is novel in identifying the gaps in mental health vulnerabilities that persist in a conflict-affected region after violence de-escalation, along with the changes in such gaps—and its possible drivers—during the first year of the COVID-19 pandemic. It provides evidence on how structural issues including economic vulnerability, social and health inequalities, and persistent barriers to health system access may have influenced mental health trends during the pandemic. Based on our findings, we discuss areas where public policy and mental health clinical services could focus on to protect the mental health of vulnerable groups, who may already suffer from mental disorders after exposure to protracted conflict violence, or who may have developed these disorders amid the pandemic [30]. Previous research has emphasized the link between poverty and mental health in LMICs, making mental health not only a public health but also a development priority [31], through policies that can effectively address the social determinants of health [32]. Of course, the most appropriate form of public policy response will depend on the specific context: our study discusses possible policy responses in a context where large population groups have suffered with decades of conflict violence, leading to mental health vulnerabilities potentially exacerbated amid the pandemic. Addressing persisting vulnerabilities generated during the Colombian conflict is still of key importance in the country, especially through better access to and quality of mental health services for groups directly affected by conflict violence [33], not least to strengthen societal support to the still fledgling peace process.

### Supplementary Information

**Additional file 1:** **Appendix S1. **The fixed-effects estimation model. **Appendix S2. **Results of the conditional fixed-effects logistic regression analysis. **Appendix S3.** Panel data selective attrition analysisTable A3.1: SRQ descriptive statistics for respondents included in and excluded from the main fixed-effects regression analysis, CONPAS 2018 survey (n=1309)Table A3.2: Tests for equality of SRQ indicators' variances and means for respondents included in and excluded from the main fixed-effects regression analysis, CONPAS 2018 survey (n=1309)

## Data Availability

The data that support the findings of this study are available upon reasonable request to the Ethics Committee at Universidad de los Andes comite-etica-investigaciones@uniandes.edu.co. The data set contains potentially identifying information from the participants.
