# Supplementary material for: Trends in mental health before and after the onset of the COVID-19 pandemic: a longitudinal survey of a conflict-affected population in Colombia
Source: Int J Ment Health Syst. 2024 Feb 5;18:4. doi: 10.1186/s13033-024-00621-1 (PMC10845752; doi:10.1186/s13033-024-00621-1)
Supplement: Supplementary file 1 — Additional file 1: Appendix S1. The fixed-effects estimation model. Appendix S2. Results of the conditional fixed-effects logistic regression analysis. Appendix S3. Panel data selective attrition analysisTable A3.1: SRQ descriptive statistics for respondents included in and excluded from the main fixed-effects regression analysis, CONPAS 2018 survey (n=1309)Table A3.2: Tests for equality of SRQ indicators' variances and means for respondents included in and excluded from the main fixed-effects regression analysis, CONPAS 2018 survey (n=1309) [file 13033_2024_621_MOESM1_ESM.docx]

**Additional file 1**

**APPENDIX 1: The fixed-effects estimation model**

The ordinary-least squares (OLS) estimator is consistent when all the relevant factors that influence the dependent variable $"y"$ are accounted for. This happens very rarely in an observational study, as there usually are unobserved explanatory variables that influence *y*. When these unobserved explanatory variables are (*i*) independent of the idiosyncratic error and (*ii*) uncorrelated with the observed explanatory variables, the OLS estimator is consistent and unbiased. However, it is often the case in observational studies that conditions (*i*) and/or (*ii*) are violated, leading to heterogeneity bias (Wooldridge, 2010).

Consider equation (1), where we disaggregate the error component *v_i,t_* into two sub-components: *a_i_*, which captures the influence of unobserved explanatory variables of *y_i,t_* that do not change over time (time-invariant factors), and *u_i,t_*, which captures the influence of other unobserved explanatory variables that vary over time:

$$y_{i,t}=\beta_{0}+\sum_{k=1}^{n} \beta_{k}X_{kit}+\delta t+v_{i,t}(1)$$

$$v_{i,t}={a_{i}+u}_{i,t}$$

$$a_{i}=\sum_{j=1}^{n} \gamma_{j}Z_{ji}$$

By performing a fixed-effects or intragroup transformation to the longitudinal data, it is possible to eliminate the influence of the time-invariant unobserved factors, or in other words to eliminate $a_{i}$. To achieve this, the first step of the fixed-effects procedure is to calculate the sample mean of each variable for each individual, for the time period under analysis. The second step is to subtract those sample means from equation 1; because *a_i_* is fixed over time, it is differentiated out in this process. This procedure leads to the “within-group” fixed-effects estimator displayed in equation (2), which explains the variations of the dependent variable around the mean as a function of the variations of the explanatory variables around their means (Wooldridge, 2010):

$$y_{i,t}-\underline{y_{i}}=\sum_{k=1}^{n} \beta_{k}{(X}_{kit}-\underline{X}_{ki})+\delta(t-\underline{t})+u_{i,t-}\underline{u}_{i}$$

$$\ddot{y_{i,t}}=\sum_{k=1}^{n} \beta_{k}\ddot{X}_{kit}+\ddot{\delta}t+\ddot{u}_{ii} (2)$$

The fixed-effects model in equation (2) addresses the unobserved heterogeneity introduced by the unobserved confounders that are constant over time. However, this model has some limitations. First, it does not allow the estimation of coefficients for variables that are constant over time such as gender or ethnicity, although these can be interacted with variables that change over time, particularly binary variables indicating the time period (e.g. year dummies), to allow marginal effects by time period to be retrieved (Wooldridge, 2010). Second, in the fixed-effects model it is not possible to estimate the effect of any variable that changes constantly over time (e.g. age), although it is possible to generate ranges of the continuous variable (e.g. age range dummies) and then include these in the model, to obtain marginal effects with respect to a base range category.

In our analysis in the main paper, the final equation representing the fixed-effects model we estimate is:

$$\ddot{y_{i,t}}=\theta+\sum_{k=1}^{n} \beta_{k}\ddot{X}_{kit}+\ddot{\delta}t+\ddot{u}_{ii} (3)$$

where the dependent variable is either the continuous SRQ score (Model 1) or the SRQ+ binary indicator (Model 2), while $\theta$ represents the time trend of the SRQ-20 score (Model 1) or of the frequency of SRQ+ cases (Model 2).

***Reference:***

Wooldridge, J. M. (2010). *Econometric analysis of cross section and panel data*. MIT press.

**APPENDIX 2: Results of the conditional fixed-effects logistic regression analysis**

|  | MODEL 3 | |
| --- | --- | --- |
|  | SRQ+ |  |
|  | ODDS RATIO (95% CI) | P>t |
| Year | | |
| 2018 (Base category) | | |
| 2019 | 0.75 (0.45; 1.28) | 0.2930 |
| 2020 | 0.30 (0.17; 0.54) | 0.0001 |
| Interaction term: Gender*Year | | |
| Women in 2018 (Base category) | | |
| Men*2019 | 1.93 (1.03; 3.61) | 0.0406 |
| Men*2020 | 2.76 (1.42; 5.36) | 0.0027 |
| Age group | | |
| 18-44 (Base category) | | |
| 45-60 | 1.66 (0.42; 6.59) | 0.4685 |
| >60 | 5.34 (0.74; 38.56) | 0.0968 |
| Household expenditure quintile | | |
| Quintile 1 (Lowest, base category) | | |
| 2 | 1.14 (0.64; 2.03) | 0.6677 |
| 3 | 1.11 (0.66; 1.87) | 0.6988 |
| 4 | 1.52 (0.87; 2.65) | 0.1435 |
| 5 (Highest) | 1.92 (1.01; 3.65) | 0.0462 |
| Employment status | | |
| Formal employee or employer (Base category) | | |
| Informal employee or self-employed | 0.60 (0.29; 1.24) | 0.1661 |
| Inactive | 0.87 (0.39; 1.92) | 0.7221 |
| Unemployed | 1.27 (0.45; 3.59) | 0.6517 |
| Retired | 5.64 (0.50; 63.85) | 0.1623 |
| Interaction term: Ethnicity*Year | | |
| Majority in 2018 (Base category) | | |
| Minority*2019 | 1.74 (0.78; 3.90) | 0.1788 |
| Minority*2020 | 2.35 (1.01; 5.47) | 0.0480 |
| Marital status | | |
| Married or stable partnership (Base category) | | |
| Separated or divorced | 2.17 (0.79; 5.98) | 0.1336 |
| Widower | 3.18 (0.43; 23.55) | 0.2583 |
| Single | 0.64 (0.09; 4.70) | 0.6650 |
| Education level | | |
| No formal education (Base category) | | |
| Preschool or primary | 1.20 (0.61; 2.32) | 0.5988 |
| High school | 1.16 (0.39; 3.48) | 0.7901 |
| Higher education | 1.01 (0.21; 4.84) | 0.9880 |
| Area of residence | | |
| Rural (Base category) | | |
| Urban | 0.64 (0.28; 1.45) | 0.2814 |
| Hospitalization in the previous 12 months | | |
| No (Base category) | | |
| Yes | 1.55 (0.92; 2.58) | 0.0967 |
| Child aged 5 or younger in the household | | |
| No (Base category) |  |  |
| Yes | 1.65 (0.78; 3.49) | 0.1879 |
| Number of people in the household | | |
| One person (Base category) | | |
| More than one person | 0.80 (0.29; 2.26) | 0.6797 |
| Conflict intensity in the municipality of residence | | |
| Not affected (Base category) | | |
| Villavicencio | 1.33 (0.06; 30.72) | 0.8574 |
| Lightly affected | 0.21 (0.01; 6.27) | 0.3662 |
| Heavily affected | 0.22 (0.01; 5.81) | 0.3683 |
| Interaction term: Displaced*Year | | |
| Not displaced in 2018 (Base category) | | |
| Displaced*2019 | 0.70 (0.38; 1.27) | 0.2396 |
| Displaced*2020 | 1.18 (0.61; 2.27) | 0.6185 |
|  | | |
| Constant | 4.04 (1.21; 6.88) | 0.0053 |

**Appendix 3: Panel data selective attrition analysis**

The first round of the CONPAS household survey included 1309 respondents. However, only 803 of these respondents participated also in the subsequent 2019 and 2020 CONPAS rounds and had a complete information set for the variables used in our study. These 803 observations constitute the actual sample that we use to conduct our fixed-effects regression analysis. In this section, we investigate whether such loss-to-follow-up is likely to have induced attrition bias in our fixed-effects analysis. This would be especially likely in our context if individuals who dropped out of the sample after the 2018 round had, on average, different mental health indicators compared to those individuals who remained in the sample for all three survey rounds (i.e. higher or lower mean SRQ scores or SRQ+ frequencies).

Using the 2018 CONPAS survey data, Table A3.1 presents the descriptive statistics of the SRQ data for those respondents who remained in the sample (“included”) and those who dropped out after the 2018 round (“excluded”). For both the SRQ score and SRQ+ indicators, the 95% confidence intervals overlap for the two groups of individuals, providing first-hand indication that mental health status was not different between the “excluded” and “included” groups at the start of the study period. We then perform in Table A3.2 (Panel A) a variance ratio *F*-test between the SRQ data of “excluded” and “included” groups: for both the SRQ score and SRQ+ indicators, we find that the null hypothesis of equality of variances between groups cannot be rejected (P-values equal to 0.582 and 0.863, respectively). Finally, in Panel B of Table A3.2 we perform *t*-tests for the difference in mean SRQ indicators between “excluded” and “included” groups. Once again, we find that the null hypothesis of no difference in means between the two groups cannot be rejected at conventional statistical significance levels, for either the SRQ score or SRQ+ indicators (P-values equal to 0.155 and 0.725, respectively).

Taken together, the results above offer reassurance that despite the loss of respondents in the 2019 and 2020 survey rounds, the respondents who remained in the sample after 2018 were not different on average from those who dropped out from the sample, with respect to their mental health status at the start of our study period. In other words, there is no evidence that those respondents who dropped out of the sample represented a selected group regarding their (better or worse) mental health status. This provides reassurance that our fixed-effects estimates are reliable in the sense that selective attrition bias is unlikely to have affected the relevant estimated coefficients.

**Table A3.1: SRQ descriptive statistics for respondents included in and excluded from the main fixed-effects regression analysis, CONPAS 2018 survey (n=1309)**

|  | Group | Observations | Mean | Standard deviation | Confidence Interval (95%) | |
| --- | --- | --- | --- | --- | --- | --- |
|  |  |  |  |  | Low | High |
| SRQ score | Included | 803 | 5.86 | 4.74 | 5.53 | 6.19 |
|  | Excluded | 506 | 5.47 | 4.84 | 5.05 | 5.90 |
|  | Combined | 1309 | 5.71 | 4.78 | 5.45 | 5.97 |
| SRQ+ | Included | 803 | 0.328 | 0.470 | 0.295 | 0.360 |
|  | Excluded | 506 | 0.318 | 0.466 | 0.277 | 0.359 |
|  | Combined | 1309 | 0.324 | 0.468 | 0.299 | 0.349 |

**Table A3.2: Tests for equality of SRQ indicators’ variances and means for respondents included in and excluded from the main fixed-effects regression analysis, CONPAS 2018 survey (n=1309)**

| *Panel A: Test for equality of variances between included and excluded groups* | | | | |
| --- | --- | --- | --- | --- |
| Null hypothesis (H0) | Alternative hypothesis (H1) | Variable | Test statistic | P-value |
| Ratio of standard deviations (included/excluded) = 1 | Ratio of standard deviations (included/excluded) ≠ 1 | SRQ score | *F* = 0.957 | 0.582 |
|  |  | SRQ+ | *F* = 1.015 | 0.863 |
| *Panel B: Test for difference in means between included and excluded groups* | | | | |
| Null hypothesis (H0) | Alternative hypothesis (H1) | Variable | Test statistic | P-value |
| Difference in means (included - excluded) = 0 | Difference in means (included - excluded) ≠ 0 | SRQ score | *t* = 1.424 | 0.155 |
|  |  | SRQ+ | *t* = 0.352 | 0.725 |
